# Psychological and sleep disturbances among first-line, second-line, and at home medical staff during the COVID-19 pandemic in Shanghai, China

**DOI:** 10.3389/fpubh.2022.1006610

**Published:** 2022-11-07

**Authors:** Ping Wang, Xiaolei Shen, Yuhan Jiang, Li Wu, Jun Shen, Xin Nie, Wei Chen, Jianren Liu

**Affiliations:** ^1^Department of Neurology, Shanghai Ninth People's Hospital, Shanghai Jiao Tong University School of Medicine, Shanghai, China; ^2^Biostatistics Office of Clinical Research Unit, Shanghai Ninth People's Hospital, Shanghai Jiao Tong University School of Medicine, Shanghai, China

**Keywords:** COVID-19, medical staff, depression, anxiety, sleep disturbances

## Abstract

**Objectives:**

Medical workers are prone to psychological and sleep disturbances during the coronavirus disease 2019 (COVID-19) pandemic. Little is known about the varying degrees of influence among vaccinated medical staff working in different positions. The current study is aimed to evaluate and compare depression, anxiety and sleep disturbances among first-line, second-line and at home vaccinated medical staff during the COVID-19 pandemic in Shanghai, China.

**Methods:**

A cross-sectional online survey was conducted in May 2022. In addition to demographic data, levels of depression, anxiety, sleep quality, and insomnia were measured using the Patient Health Questionnaire-9 (PHQ-9), Generalized Anxiety Disorder-7 (GAD-7), Pittsburgh Sleep Quality Index (PSQI), and Athens Insomnia Scale (AIS).

**Results:**

A total of 236 vaccinated medical workers completed the questionnaires, including 85 first-line medical staff (FMS), 82 second-line medical staff (SMS) and 69 at home medical staff (HMS). The proportions of depressive symptoms, anxiety symptoms, poor sleep quality, and insomnia were 52.1, 44.1, 55.9, and 49.2%, respectively. Compared with HMS, medical staff at work (FMS and SMS) got significantly higher frequency of poor sleep quality (both *p* < 0.001), insomnia (both *p* < 0.001), depressive (*p* < 0.001 and *p* = 0.003, respectively) and anxiety symptoms (*p* < 0.001 and *p* = 0.002, respectively). Compared with SMS, FMS were more likely to have poor sleep quality (*p* = 0.020). Besides, nurses got significantly higher percentage of poor sleep quality (OR = 1.352, *p* = 0.016) and insomnia (OR = 1.243, *p* = 0.041) than doctors. Whereas, the proportion of anxiety symptoms was increased in females than in males (OR = 2.772, *p* = 0.008).

**Conclusions:**

Psychological and sleep disturbances are common among medical staff at work during the COVID-19 pandemic. More psychological intervention should be administrated for FMS, especially for nurses.

## Introduction

Since the first case of coronavirus disease 2019 (COVID-2019) was detected in December 2019 in Hubei province of China, it caused a pandemic in the world (1). COVID-2019 is a highly contagious disease with asymptomatic infection during the incubation period, and can be transmitted through respiratory droplets, contact, and aerosols (2). In late February 2022, a wave of mutated coronavirus infection named omicron quickly spread throughout Shanghai, China. Within three months, more than 600,000 people were reported with omicron infection in Shanghai, China (3). With the rapid reconstruction of mobile cabin and designated hospitals, more and more medical staff joined the team to combat the pandemic. Due to the rapid spread of the virus variants and high risk of infection, medical staff are under enormous psychological pressure, which may lead to various disorders such as anxiety, depression, and insomnia. In previous studies since 2020, the psychological pressure brought by the outbreak of COVID-2019 to medical staff has been paid attention to Kang et al. (4), Kang et al. (5), Korkmaz et al. (6), Xiao et al. (7), and Zhang et al. (8). A recent meta-analysis by Aymerich et al. reported frequencies of 33% for depressive symptoms, 42% for anxiety and 42% for insomnia (9).

Until February 2022, the majority of medical staff had got at least two doses of COVID-19 vaccine, which may protect them from serious infection while treating COVID-19 patients. Compared with the Wuhan epidemic in 2020, the omicron variant of COVID-19 in Shanghai spread more rapidly, but with less virulent and lower fatality rate (2). Based on the above characteristics, will the psychological conditions of medical staff in Shanghai be different from those in Wuhan or other countries? Until now, there have been no study on the psychological pressure to vaccinated medical staff working in different positions in Shanghai, China. Therefore, in this study, we aim to compare depression, anxiety, and sleep disturbances among vaccinated first-line, second-line, and at home medical staff during the COVID-19 pandemic in Shanghai, China.

## Materials and methods

### Participants

The cross-sectional study included medical staff who have worked in Shanghai in May 2022. This survey was conducted anonymously. The inclusion criteria were as follows: (a) age ranged from 18 to 65; (b) got at least two doses of COVID-19 vaccine; (c) medical workers in Shanghai; (d) volunteered to participate in the survey. Having a history of certain mental and physical disorders and previous taking sleep modulating medication were considered as the exclusion criteria. Participants were divided into three groups: first-line medical staff (FMS), second-line medical staff (SMS), and at home medical staff (HMS) depending on whether they worked and were directly exposed to COVID-19 patients in current working position. FMS worked in mobile cabin hospitals or designated hospitals specialized for COVID-19 patients, SMS worked in general departments, including clinic, emergency and wards, while some medical staff stayed at home because of community containment. This study was approved by the ethics committee of Shanghai Ninth People's Hospital, Shanghai Jiao Tong University School of Medicine (Ethical number: SH9H-2022-T113-1).

### Data collection

The online survey was conducted in May 2022. In addition to demographic and general data (age, gender, education years, department, seniority, working position, caffeine intake, tobacco use, exercise, etc.), we mainly collected Pittsburgh Sleep Quality Index (PSQI), Athens Insomnia Scale (AIS), Patient Health Questionnaire-9 (PHQ-9) and Generalized Anxiety Disorder-7 (GAD-7).

### The Pittsburgh sleep quality index (PSQI)

PSQI is a self-reported questionnaire which evaluates subjective sleep quality within 1 month using an 18-item scale containing seven items, including sleep quality, sleep latency, sleep duration, habitual sleep efficiency, sleep disturbance, use of sleeping medication, and daytime dysfunction. Each dimension is weighted on a scoring scale between 0 and 3, and the scores of these seven sub-dimensions are summed up to determine the total score, ranging from 0 to 21. The higher the PSQI, the worse the sleep quality (10). Scores >6 indicate a disturbance in sleep quality (11). The Chinese version of the PSQI was translated in 1996 by Liu et al. and have been examined in different populations, all indicating that it is a reliable and valid instrument for evaluating sleep quality (12).

### Athens insomnia scale (AIS)

The AIS is a brief instrument to assess the severity of insomnia. It contains eight items with each item rated from 0 to 3. The total score ranging from 0 to 24 was included in the analysis. The higher the AIS, the more severe the insomnia (13). A subject with a total score>6 points was considered positive for insomnia (11). The Chinese version of this scale was proved to be a reliable and valid instrument (14).

### Patient health questionnaire depression module-9 (PHQ-9)

The PHQ-9 is a self-rated module for measuring the levels of depression. It was previously translated into Chinese and validated by researchers in China (15). It consists of nine questions relating to the patients' mental health, with each item rated on a 4-point scale. An aggregate score of nine was used in our study, with higher scores indicating more severe level of depression (16). A score>4is identified as having depressive symptoms (17).

### Generalized anxiety disorder-7 (GAD-7)

The GAD-7 is a self-rated screening tool for possible anxiety disorders. It consists of seven items, with a score for each item of between 0 and 3, and the total score of 0–21. The GAD-7 showed good reliability and validity in a Chinese population (18). In this study, the total score of the questionnaire was taken into consideration. Higher scores of total GAD-7 indicated stronger feeling of anxiety (19). A score >4 is identified as having anxiety symptoms (17).

### Statistical analysis

Statistical analyses were performed using SPSS 25.0 for Windows. Categorical variables were described as percentages, and continuous variables were described as mean ± standard deviation (SD). Overall differences among the groups were conducted by Chi-square test or Fisher's exact test for categorical data. For continuous variables, one-way analysis of variance (ANOVA) or Kruskal-wallis test was used depending on whether or not the variables are normally distributed. The least significant difference (LSD) method was adopted for *post hoc* analysis.

Binary logistic regression analyses were used to explore the associated factors of depressive, anxiety symptoms and sleep disturbances, variables with *p* < 0.05 in univariate logistic analyses were included in the multivariate logistic regression analyses. Spearman correlation analysis was utilized to explore the bivariate correlation among the four questionnaires. The level of significance for all comparisons was set at *p* < 0.05.

## Results

### Demographic characteristics

Two hundred and thirty-six medical staff volunteered to answer the online survey and all of them (85 FMS, 82 SMS, and 69 HMS) were finally enrolled in the study. The detailed demographic features are shown in Table 1. Three groups were matched on gender, years of education, profession, working years, tobacco use and exercise. However, FMS were younger and had less caffeine intake than SMS and HMS.

**Table 1:** Demographic data of the enrolled subjects.

	**HMS (*n* = 69)**	**SMS (*n* = 82)**	**FMS (*n* = 85)**	***P***	
				**Global**	**SMS vs. HMS**
					**FMS vs. HMS**
					**FMS vs. SMS**
Age, mean (SD)	38.3 (8.5)	39.8 (7.9)	33.4 (7.6)	< 0.001^***^	0.265
					< 0.001^***^
					< 0.001^***^
Male, *n* (%)	15 (21.7)	16 (19.5)	20 (23.5)	0.819	Na
Years of education, mean (SD)	19.0 (2.9)	18.9 (3.1)	18.1 (2.7)	0.182	Na
Doctor, *n* (%)	26 (37.7)	32 (39.0)	31 (36.5)	0.149	Na
Working years, mean (SD)	9.1(5.3)	9.9 (3.9)	8.3 (4.8)	0.061	Na
Caffeine intake, *n* (%)	52 (75.4) ^**^	58 (70.7) ^**^	43 (50.1)	0.002^**^	0.524
					0.002^**^
					0.008^**^
Tobacco use, *n* (%)	2 (2.9)	2 (2.4)	6 (7.0)	0.238	Na
Exercise, *n* (%)	40 (58.0)	50 (60.9)	49 (57.6)	0.887	Na

FMS, first-line medical staff; SMS, second-line medical staff; HMS, at home medical staff.

** p < 0.01,

*** < 0.001.

### Associated factors of psychological and sleep disturbances in medical workers

As shown in Table 2, occupation and educational level were closely associated with sleep disturbances. Nurses got significantly higher percentages of poor sleep quality (OR = 1.352, *p* = 0.016) and insomnia (OR = 1.243, *p* = 0.041) than doctors, and low education level was a risk factor for poor sleep quality (*p* < 0.001) and insomnia (*p* = 0.004). Besides, female medical staff were more likely to experience anxiety symptoms than males (OR = 2.772, *P* = 0.008). However, other factors such as age, working experience, caffeine intake, tobacco use, and exercise had little effects on the occurrence of depressive symptoms, anxiety symptoms, and sleep disturbances.

**Table 2:** Logistic regression analyses for associated factors of psychological and sleep disturbances.

	**Poor sleep quality**		**Insomnia**		**Depressive symptoms**		**Anxiety symptoms**	
	**OR (95%CI)**	***P***	**OR (95%CI)**	***P***	**OR (95%CI)**	***P***	**OR (95%CI)**	***P***
Age	0.992 (0.955, 1.030)	0.673	0.969 (0.933, 1.007)	0.106	0.969 (0.934, 1.006)	0.103	1.011 (0.975, 1.049)	0.552
Gender (Female)	1.225 (0.608, 2.469)	0.570	1.757 (0.864, 3.573)	0.120	1.435(0.725, 2.839)	0.299	2.772 (1.301, 5.905)	0.008^**^
Profession (Nurse)	1.352 (1.184, 2.285)	0.016^**^	1.243 (1.021, 2.165)	0.041^*^	0.761 (0.438, 1.324)	0.334	1.022 (0.579, 1.803)	0.941
Years of education	0.777 (0.682, 0.885)	< 0.001^***^	0.836 (0.739, 0.944)	0.004^**^	0.928 (0.826, 1.042)	0.207	1.017 (0.903, 1.146)	0.778
Working years	0.985 (0.921, 1.054)	0.664	1.050 (0.981, 1.124)	0.159	1.059(0.990, 1.132)	0.098	1.018 (0.952, 1.088)	0.606
Caffeine intake	0.698 (0.390, 1.251)	0.227	0.926 (0.525, 1.634)	0.792	1.141 (0.654, 1.991)	0.642	1.233 (0.701, 2.169)	0.467
Tobacco use	4.348 (0.864, 21.880)	0.075	2.195 (0.546, 1.634)	0.268	1.378 (0.363, 5.226)	0.637	2.675 (0.678, 10.557)	0.160
Exercise	0.949 (0.672, 1.340)	0.767	1.034 (0.734, 1.455)	0.850	0.950 (0.678, 1.329)	0.763	0.805 (0.571, 1.136)	0.218

Based on univariate analyses of binary logistic regression model.

* p < 0.05,

** p < 0.01,

*** p < 0.001.

**Figure 1:**
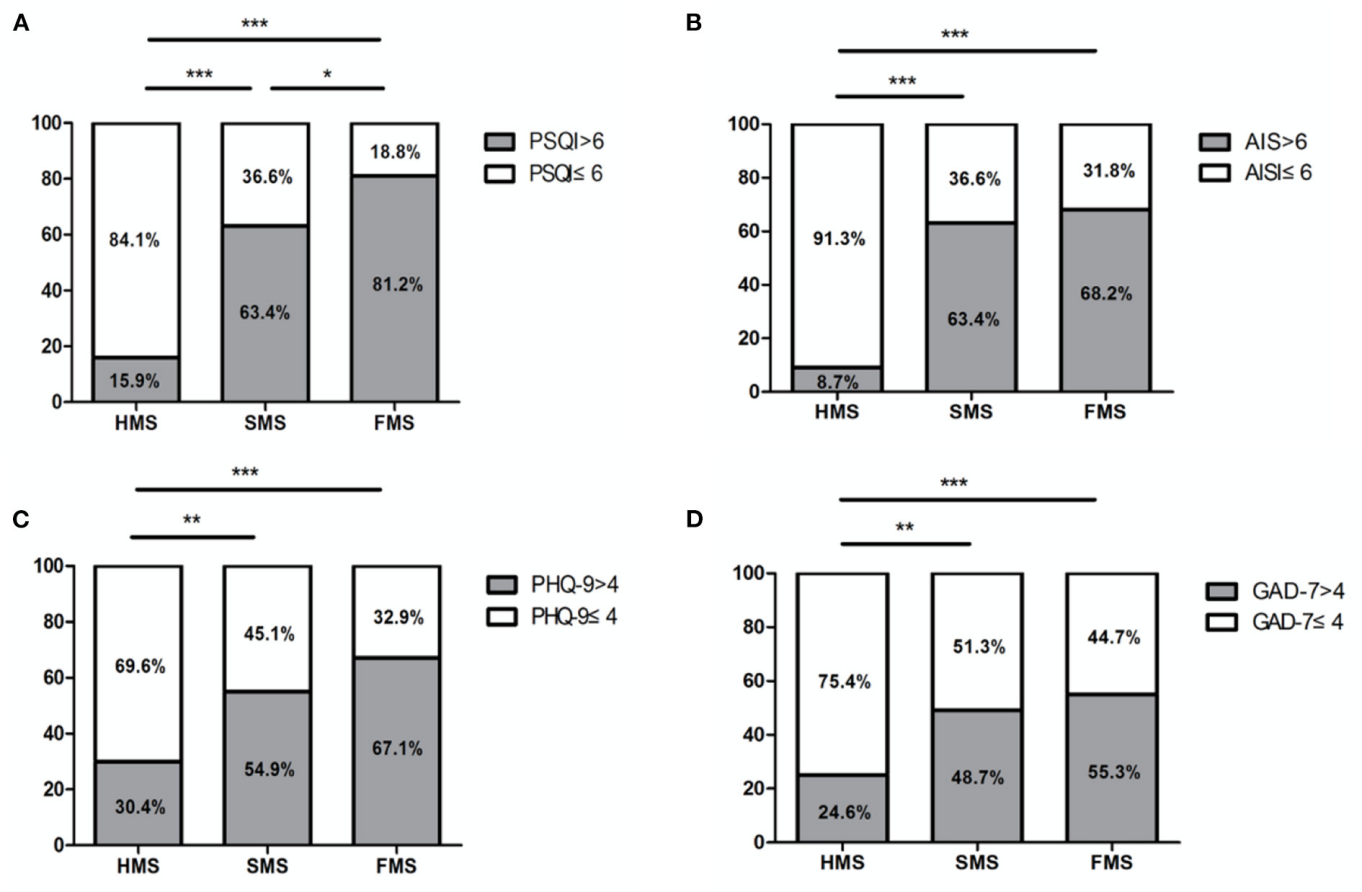
Proportion of poor sleep quality, insomnia, depressive symptoms, and anxiety symptoms among HMS, SMS and FMS. **(A)** The FMS and SMS had significantly higher proportion of poor sleep quality than HMS, the FMS had significantly higher proportion of poor sleep quality than SMS. **(B)** The FMS and SMS had significantly higher proportion of insomnia than HMS. **(C)** The FMS and SMS had significantly higher proportion of depressive symptoms than HMS. **(D)** The FMS and SMS had significantly higher proportion of anxiety symptoms than HMS. FMS, first-line medical staff; SMS, second-line medical staff; HMS, at home medical staff; PSQI, Pittsburgh Sleep Quality Index; AIS, Athens Insomnia Scale; PHQ-9, Patient Health Questionnaire depression module-9; GAD-7, Generalized Anxiety Disorder-7; *p < 0.05, **p < 0.01, ***p < 0.001.

After adjusting for age, gender, profession, educational year and caffeine intake, binary logistic regression analyses revealed that workplace during the COVID-19 pandemic was an independent influencing factor of psychological and sleep disturbances. Compared with HMS, FMS and SMS got significantly higher percentage of poor sleep quality (both *p* < 0.001), insomnia (both *p* < 0.001), depressive (*p* < 0.001 and *p* = 0.003, respectively) and anxiety symptoms (*p* < 0.001 and *p* = 0.002, respectively; Figure 1 and Table 3). Compared with SMS, FMS got significantly higher proportions of poor sleep quality (*p* = 0.020) (Figure 1).

**Table 3:** Logistic regression analyses of psychological and sleep disturbances stratified by working position.

	**Poor sleep quality**			**Insomnia**			**Depressive symptoms**			**Anxiety symptoms**		
	***n* (%)**	**OR (95%CI)**	***P***	***n* (%)**	**OR (95%CI)**	***P***	***n* (%)**	**OR (95%CI)**	***P***	***n* (%)**	**OR (95%CI)**	***P***
HMS	11 (15.9)	Ref		6 (8.7)	Ref		21 (30.4)	Ref		17 (24.6)	Ref	
SMS	52 (63.4)	9.490 (4.195, 21.464)	< 0.001^***^	52 (63.4)	18.908(7.199, 49.660)	< 0.001^***^	45 (54.9)	2.815 (1.422, 5.572)	0.003^**^	40 (48.8)	3.080 (1.489, 6.370)	0.002^**^
FMS	69 (81.2)	24.779 (9.977, 61.741)	< 0.001^***^	58 (68.2)	24.111(8.805, 66.025)	< 0.001^***^	57 (67.1)	5.186 (2.469, 10.892)	< 0.001^***^	47 (55.3)	6.077 (2.743, 13.463)	< 0.001^***^

FMS, first-line medical staff; SMS, second-line medical staff; HMS, at home medical staff.

Confounding factors (age, gender, profession, years of education and caffeine intake) were entered into the binary logistic regression analysis model.

** p < 0.01,

*** p < 0.001.

### Correlation analyses among the four questionnaires

The levels of depression, anxiety, sleep quality and insomnia were measured using PHQ-9, GAD-7, PSQI, and AIS. Spearman correlation analysis was used to identify the correlations between the results from the responses of the medical staff. As demonstrated in Table 4, there was a significant positive correlation between each of the four questionnaires.

**Table 4:** Correlations among the four questionnaires.

	**PSQI**	**AIS**	**PHQ-9**	**GAD-7**
PSQI	1	0.854^**^	0.662^**^	0.520^**^
AIS	0.854^**^	1	0.717^**^	0.565^**^
PHQ-9	0.662^**^	0.717^**^	1	0.752^**^
GAD-7	0.520^**^	0.565^**^	0.752^**^	1

PSQI, Pittsburgh Sleep Quality Index; AIS, Athens Insomnia Scale; PHQ-9, Patient Health Questionnaire depression module-9; GAD-7, Generalized Anxiety Disorder-7.

Spearman correlation coefficient was presented in this table.

^**^p < 0.01.

## Discussion

Previous studies have found that medical staff were vulnerable to mental health problems during the COVID-19 pandemic, because of the high risk of exposure and infection due to their profession (7, 11). However, there was no study to compare these mental health problems among first-line, second-line, and at home vaccinated medical staff. This study is the first one to investigate the levels of depression, anxiety, and sleep disturbances among vaccinated medical staff in different positions during the COVID-19 pandemic in Shanghai, China. We mainly found that (1) the percentages of depressive symptoms, anxiety symptoms and sleep disturbances of medical staff at work (FMS and SMS) were higher than those at home (HMS); (2) FMS had higher proportions of poor sleep quality than SMS; (3) Profession and educational year were closely associated with sleep disturbances, and females were more likely to experience anxiety symptoms than males; (4) There were significant correlations among the four scales of PHQ-9, GAD-7, PSQI, and AIS.

We found that the percentage of depressive symptoms, anxiety symptoms, and insomnia were 61.1, 52.1, 65.9%, respectively, among medical staff at work in the present study. Lai et al. reported the prevalence of depression, anxiety and insomnia in the total medical staff were 50.4, 44.6, and 34.0% (20), which were lower than our results. The reason may be that they did not distinguish between medical staff at work and those at home. Medical staff who were not treating directly the infected or potentially infected patients had less psychological distress. In addition, FMS had higher frequency of poor sleep quality than SMS, which may be due to the added stress that FMS were required to wear protective masks and clothing. Therefore, medical staff at work especially FMS were under unprecedented mental distress, thus it is necessary to provide mental health support to these people.

In the present study, we found that nurses were more likely to experience poor sleep quality and insomnia than doctors during the COVID-19 pandemic, which is in accordance to the results of the previous studies (21, 22). This could be explained by the factor that nurses are mostly female and responsible for the collection of sputum for virus detection. They spend more time and provide direct care to COVID-19 patients (20, 23). Besides profession, we found that low education level was also a risk factor for poor sleep quality and insomnia. This result is consistent with the report from Schou-Bredal et al. in Norway (24). One possibility is that FMS with a high educational level have more professional understanding of COVID-19 and are less afraid of it. Besides, nurses are usually less educated than doctors. Therefore, nurses need more mental health support during the COVID-19 pandemic.

In accordance with the results of previous studies (25, 26), the findings from this study showed that there was a bidirectional relationship between depression, anxiety and sleep disturbances, indicating that each factor could contribute to the development or is a consequence of another one. The potential mechanisms could be attribute to the common neurobiological underpinnings (i.e., altered neurotransmitters and brain functions) and increased inflammatory dysregulation in depression, anxiety, and sleep disturbances (27). Therefore, public health education and clinical interventions for each disorder is essential for medical workers, especially for FMS. Successful intervention of sleep disturbances may prevent the onset of subsequent or exacerbation of comorbid anxiety or depression, and vice-versa (26).

A few limitations should be mentioned in this study. First, this was a cross-sectional study with a limited sample size. Second, all participants volunteered to this study, which could cause selection biases. Third, we used self-reported questionnaires to obtain the data, solely based on subjects' reaction or opinion. However, the findings in this pilot study are significant. Psychological and sleep disturbances are common among medical staff at work during the COVID-19 pandemic. This has important implications for public health. More psychological intervention should be administrated for FMS, especially for nurses. A longitudinal follow-up study with a large sample size is warranted in future to investigate the nature course of psychological and sleep disturbance among medical workers after the COVID-19 pandemic.

## Data availability statement

The raw data supporting the conclusions of this article will be made available by the authors, without undue reservation.

## Ethics statement

The studies involving human participants were reviewed and approved by this study was approved by the Ethics Committee of Shanghai Ninth People's Hospital, Shanghai Jiao Tong University School of Medicine (Ethical number: SH9H-2022-T113-1). The patients/participants provided their written informed consent to participate in this study.

## Author contributions

PW, YJ, WC, and JL designed the study. PW, XS, XN, and WC performed the data analysis. PW, YJ, XS, JS, LW, WC, and JL participated in the collection and interpretation of data. PW wrote the paper with input from all authors. All authors discussed the results and contributed to the final manuscript, revised the manuscript content, and approved the final version of the manuscript.

## Funding

This research was supported by grants from 200 talent project from Shanghai Municipal Education Commission-Gaofeng Clinical Medicine Grant Support (20161422 to JL), Natural Science Foundation Project from the Shanghai Municipal Science and Technology Commission (22ZR1436900 to JL), Clinical Research Program of Ninth People's Hospital affiliated to Shanghai Jiao Tong University School of Medicine (JYLJ202003 to WC), and Project of Biobank from Shanghai Ninth People's Hospital, Shanghai Jiao Tong University School of Medicine (YBKB202120 to WC).

## Conflict of interest

The authors declare that the research was conducted in the absence of any commercial or financial relationships that could be construed as a potential conflict of interest.

## Publisher's note

All claims expressed in this article are solely those of the authors and do not necessarily represent those of their affiliated organizations, or those of the publisher, the editors and the reviewers. Any product that may be evaluated in this article, or claim that may be made by its manufacturer, is not guaranteed or endorsed by the publisher.
